# Notes and Comments

**Published:** 1924-11

**Authors:** 


### NOTES AND COMMENTS.

### CO-EDUCATION AND ST. MARY'S.

THE authorities of St. Mary's Hospital have finally decided to follow the example of the London Hospital and to exclude for the future women medical students from the hospital and school. There has been an unfortunate and widespread belief that the real reasons for a decision adverse to the women students are never made public. The effect, whatever the men's hospitals do, has been to convert the controversy into a poison which affects the hospitals, the medical profession, the students of both sexes, and especially the outside public in many disastrous ways. Courage and public spirit have too often been lacking for the statement, in all frankness, of the arguments for and against co-education in medicine. Yet such a statement would have been the best way of clearing the air. Co-education can never be a simple matter, whatever fanatics on either side may say. The truth would not be the vital thing it is if it were not liable to upset ill-balanced minds. But timidity is not the same thing as discretion, and on certain pressing matters, of which medical co-education happens to be one at the moment, the better part of policy is to be perfectly frank. The decision at St. Mary's Hospital is stated to have been made on financial grounds only. We trust, therefore, that the public mind will now be set at rest and the controversy lapse accordingly.

### IMPROVING HEALTH WEEK.

INDICATIONS are not lacking that the recent prize competition in THE HOSPITAL AND HEALTH REVIEW for the best method of observing Health Week has had its effect. Not only was the Week kept more widely than before, but its concomitants were more ingenious, more interesting and more practical. Especial stress was laid this year upon the importance of sunlight as a preventive and cure of disease, and the consequent need of clearing the atmosphere of the smoke which forbids heat and light to reach us. Much, too, was heard of the urgent need of reducing the mortality among children of from one to five years of age, and of the care of the teeth from childhood. These are among the most vital subjects connected with the public health, and they are subjects upon which the national attention has only of late become concentrated. That Health Week has exercised a powerful influence upon the public mind is beyond question, but it is essential that it should not become stereotyped, but should be kept fresh and novel and annually strike some fresh note, while not forgetting the necessary play upon the old ones. Health Week is at once an occasion for enlightened selfishness and for brilliant altruism. In guarding our own health we guard that of the community—we set a good example which at the same time helps ourselves, as a good example always does, however unconsciously. Very soon there ought not to be a town or village in the country which lacks its annual opportunity of emphasizing the duty of keeping ourselves and our neighbours healthy.

### SPIRITUAL HEALING.

DURING the past month events at Bradford and elsewhere have once more brought spiritual healing prominently before us. We hear, as usual, of cripples throwing away their crutches and "permanent invalids" being "cured" on the spot, of congregations in churches worked up to a high degree of spiritual exultation, and of clergy with tears streaming down their faces. Ecstatic emotionalism is an inevitable accompaniment of such scenes, and we must strip them of these accompaniments before we can form a reasoned judgment of what may be supposed really to have happened. We are far, indeed, from suggesting that human ills can never be cured by spiritual means—we know too little to deny the possibility with safety and too much to take up an attitude of irreconcilable scepticism. But when we are asked to accept what are described as "miraculous" cures, we are often tempted to say either that the supposed disease did not, as a fact, exist, or that the patient was not cured. It is in the highest degree unfortunate that spiritual healing, so called, has for centuries been closely mixed up with proved charlatanism, and we must clear our minds of the prejudice thus created before we can bring ourselves to a dispassionate consideration of a subject of great and solemn importance. We hear far too little of the after-history of the "cured" on occasions such as that at Bradford, and are consequently deprived of the evidential help which is essential to calm judgment. Meanwhile we note with great satisfaction that Mr. J. M. Hickson, after his remarkable experiences at Bradford once more definitely refuses to claim any healing powers of his own. We shall hope to have some definite medical report of the condition of the "cured" at Bradford, say, six months hence.

### THE BEST INVESTMENT.

THE Parliamentary Secretary of the Ministry of Health in referring, in the course of a Health Week talk at Stepney, to the considerable decline in the general death-rate, and especially in the infant mortality rate in recent years in Stepney, said that, with its special difficulties due to its restricted area and dense population, this was a remarkable testimony to the work of the public health service. He paid a high tribute to the local maternity and child welfare work, and emphasised the importance of the ante-natal clinic and the care of mothers and children. Of the country generally, the Parliamentary Secretary referred to the enormous loss of time through sickness, emphasising the fact that boys and girls carried with them, on entry into industrial life, a mass of disability. He pointed out that the healthier the lives of mothers and children, the less would be the mass of disease and disablement in after-life. At present, three thousand women lose their lives in childbirth each year and one out of every 14 infants dies before it is a year old. There could be no better national investment than healthy motherhood and childhood.

### HEALTHY LONDON.

THAT London is one of the healthiest places in the world is a truism, and the annual report upon its health, which has just been issued by the County Council, shows that, so far as longevity and immunity from disease are concerned, it improves steadily. The Metropolitan death-rate has been continuously declining for the last fifty years, and the decline is especially marked at school ages. It is now 35 per cent. less than in 1841. The extent to which the health of London is affected by the constant influx of the provincial born would form a curious topic for speculation—very nearly one-half of the population of the so-called "County of London" were born far from the sound of Bow bells. For a good many years past it has been noticed that the English complexion tends to grow darker, and the report draws attention to the singular circumstance that dark people are more adaptable to the conditions of town life than the blond type. Welshmen, Jews, and Italians are singled out as showing adaptability to urban life definitely superior to that of the lighter races—that is to say, they possess greater powers of resistance. We can hardly doubt that Greater London shows similar phenomena, since much the same conditions exist in the outer zone as in the artificially created "Administrative County of London." Is the hereditary Londoner—the cockney born and bred—preponderatingly dark in hue? It is obvious that he ought to be.

### "IGNORANT ÆSTHETICISM."

DR. FRIEND, the Medical Officer of Christ's Hospital, deserves thanks for inventing a useful phrase with which to belabour people who persist in demanding artificially white bread. They are, he says, the victims of an "ignorant æstheticism." We feel more sure of the ignorance than of the æsthetics. As Sir Harry Baldwin told the Medical Officers of Schools Association, which has been discussing this very important subject, the liking for white flour is a "vitiated taste," and Dr. Friend gave a notable instance of the advantages of stone-milled or wholemeal bread. The Christ's Hospital boys have for twenty-two years been eating bread made of 76 to 80 per cent. flour. In the first year the average of carious teeth per boy was four; now it is only a fraction over one. Real bread ensures proper mastication, and Sir Harry Baldwin is inclined to attribute the smallness of the modern jaw to the removal of vitamins from flour. It is peculiarly absurd and highly mischievous that we should for so long have used the word "offal" to describe those vital constituents of flour which we mill away and use for feeding live stock. We are sometimes assured that millers and bakers would be quite ready to give us nourishing flour and well-baked bread if only the public demanded these things. We do not believe it. So long as it is more profitable to produce pasty and ill-baked bread, that is the bread we shall have to eat, failing, of course, a general rising of the better educated part of the population. But it is perhaps a mistake always to demand wholemeal bread. What is really needed is the return of the palatable and nutritious "Standard bread" introduced by the late Sir Oswald Mosley.

### FIRST YEAR'S WORK OF MAUDSLEY HOSPITAL.

THE pioneer work of the Maudsley Hospital is of such interest and value that the first annual report of its medical superintendent, Dr. Mapother, is worthy of careful study. The hospital has treated over a thousand patients, which is an encouraging record for its first year, and an almost equal number of applications were received which ended without the patients being seen because for various reasons they were ineligible. The majority of cases were sent by general practitioners who are clearly glad of the chance to confide patients whom they have neither time, nor perhaps qualifications, to treat, to a specialist institution. There are now few spontaneous applications, though upon the opening of the hospital a fair number were forthcoming. As was to be expected many of these applicants were chronic neurotics. Hospitals provide a steadily increasing stream, but the number sent on from poor-law infirmaries and observation wards has been distinctly disappointing since, as Dr. Mapother points out, a "very large number of entirely suitable patients suffering from various psychoses are annually certified there and sent to the mental hospitals of the county." The out-patient department is stated to be an "unquestionable success" and disposes of the argument that patients with minor mental disorders would be unwilling to attend the out-patient clinic at a mental hospital, and that such clinics should be developed at general hospitals. Indeed Dr. Mapother, who is also in charge of a similar department at King's College Hospital, finds, with regret, that "the opening of the Maudsley has much reduced the formerly considerable attendance at King's." There is no other institution in England on the lines of the Maudsley Hospital, and we trust that it will become increasingly well known and supported by medical men in every sphere of work.

### DIVIDED COUNCILS.

WHEN a man consults a lawyer or a doctor and fails to follow his advice, there are usually only two explanations of his refusal—either he is a fool or he has no confidence in his adviser. We have no desire to be rude to Mr. Wheatley, but it is impossible not to wonder why he takes one line about vaccination and his Chief Medical Officer an entirely opposite one. Very recently Sir George Newman in his annual report declared that "the public has taken dangerous advantage" of the facilities for obtaining exemption from vaccination, yet eleven days later the Minister of Health issued an order increasing those facilities. Although he has been questioned on the subject in the House of Commons, Mr. Wheatley has refused explanations. Can it be that, in this case, there is a third alternative—that of politics? Of all imaginable public departments that to which is confided the health of the country ought to stand most severely aloof from politics in the narrow sense of the word. Unhappily vaccination, like housing, has a political aspect to which we fear Mr. Wheatley has not been insensible. In any case, the country may reasonably expect its Ministry of Health to speak with one voice upon such a subject as disease and the means of preventing it. We know that vaccination has been the principal agent in reducing both the prevalence and the virulence of small-pox, and in a matter of such moment we cannot afford to put back the clock.

### HOSPITALS AND BLINDNESS.

WE have dealt at large elsewhere with Dr. Kay Menzies's report on the voluntary hospitals, but there is one portion of that admirable document to which separate attention ought to be called. Dr. Menzies says, most truly, that it is "almost a national scandal" that Ophthalmia Neonatorum should still be responsible for approximately 30 per cent. of all cases of total blindness in this country, not to mention a large number of cases of partial blindness. The provision for its treatment is inadequate, and he finds it "difficult to imagine any appeal more likely to be responded to than one designed to provide sufficient hospital facilities for the treatment of this acute, highly dangerous and infectious disease." Yet it is eminently curable, and the hospitals are the proper means for curing it. Not only is it readily curable in the early stages but readily preventable, given the means of prevention. Blindness is so terrible a tragedy that if we can prevent it and fail to do so we are misusing our opportunities and depriving our hospitals of a peculiarly touching and effective claim to public support. They cannot afford, by remaining unresponsive to this call, to leave open the way for the Public Health Authorities to step in and perform a great act of prevention and cure.

### APT ADULTERATION'S ARTFUL AID.

THE statistician in search of records has beome a familiar object, and of late he has often had welcome news. We make lowered records in death-rates, in infant mortality and, unhappily, in marriages. Now we are making them in adulteration. The Blue Book on food adulteration which has just been issued shows that the percentage of all-round adulteration was last year 6.1 per cent—the smallest yet. It is true that it is the merest fraction less than in 1922, but in such a matter as this even fractions count. That London methods of inspection are more efficient than those of the provinces is suggested by the superiority of the metropolitan figures. The percentage of adulteration in the capital was only 4.6 as against 7.7 in towns with populations not exceeding 100,000. The adulterator naturally turns to milk—we have learned not to expect exaggerated purity in that direction. Sometimes his methods are artful, but quite as often they are crude. Thus in one case milk was endowed with consistency by the addition from a dirty cattle trough of water which contained "myriads of micro-organisms, green deposits and fungus growths." The person who performed this operation was indulged with a fine of £10. Many samples of milk contained boric acid, the use of which, as we have shown in our second leading article this month, has just been strongly condemned by the Departmental Committee on Food Preservatives. Not all adulteration is harmful, but it is always dishonest, as when the makers of jam "improve" a comparatively dear variety by adding cheaper fruits or their juices. The way of all these transgressors is harder than it used to be, but we may hope that it will be made much harder in the future.

### EXAMINING POLICY HOLDERS.

WE notice that an insurance company has recently offered to arrange, free of all charge, for a thorough annual medical overhaul of its policy-holders, and, as various provisions are made to remove those factors which might deter them from accepting this invitation, it is clear that one more good move has been made towards systematic detection of the beginnings of disease as opposed to attempted cure of developed conditions only. America is far ahead of us in these matters. There, every possible means is being taken to stimulate popular interest in the value of periodic health examinations. Lectures, motion pictures, numberless committees and every conceivable aid to an extensive publicity campaign are being brought into play and the medical profession is an obviously active partner in the development. All this is in marked contrast with the apparent apathy with which the matter, though freely discussed, is dealt with in England. We can only hope that this most obvious course in practical preventive medicine will soon surely, if slowly, become customary on this side of the Atlantic also.

### HEALTH AND PENMANSHIP.

THE German graphologists have been holding a conference at which they have claimed that the delineation of character from handwriting is a real and not a pseudo-science, and have expressed their conviction that before long German Medical Faculties will find it desirable to establish Chairs of Graphology. They have, in fact, found a doctor in a university clinic ready to declare that the handwriting of a patient is a help in the diagnosis of disease! Nor is he content to stop there, but goes on to urge that medical post-graduates should be given a special course in graphology. We can readily imagine that these young gentlemen who have been compelled to learn so many things for so many years might be tempted to stab this troublesome person with a poisoned pen—it is bad enough to have to acquire all manner of real sciences without having a doubtful one superadded. If, however, we could be sure that handwriting was a real test of health, its study might save both doctor and patient a vast amount of trouble. Certain individual characteristics can unquestionably often be deduced from the handwriting of educated persons—precision, caution, buoyancy, or the reverse, ideality, determination, for instance. But what are the calligraphic marks that are to assure us that the writer is subject to headaches, or gout, or housemaid's knee? And what should be the peculiar formations that would suggest deep-seated latent disease ? We must not expect too much self-revelation from the nervous movements of the hand that holds a pen, and it is useful to remember that it was a German professor who derived his notion of a camel from his inner consciousness.

### CONGESTED ASYLUMS.

ACCORDING to Sir Frederick J. Willis, the Chairman of the Board of Control, the remaining accommodation in our mental hospitals will be used up in twelve months, not by the increase of insanity, but by the growth of population, and the statement is supported by the Board's since issued annual report, which shows a heavy increase in the number of lunatics. That is one of the most outstanding statements that have yet been made to the Royal Commission on Lunacy Law and Administration, and the Board suggests that, to meet the difficulty, fuller use should be made of workhouse accommodation. It would like to see all workhouse and mental hospital accommodation placed under the control of a Visiting Committee, or, failing that, that the Visiting Committees should be in a position to supervise and direct the operations of the Guardians in so far as mental patients are concerned. It would thus appear that the county mental hospitals are suffering from the same kind of congestion as the voluntary hospitals, and that a very similar remedy is proposed. The Commission has as yet got little beyond the fringe of the very serious subject into which it is inquiring, but the public will note with anxiety Sir Frederick Willis's statement that the private mental patient is better protected than the man who is looked after at the public expense because " there are more formalities to be observed." The vast majority of certified persons are in public institutions, and the majority ought to enjoy protection as full as that given to the minority.

### ARTIFICIAL LIGHT TREATMENT.

IN an interesting article in this issue we refer to the wonderful work being performed by sunshine and open air in the Alpine treatment of children suffering from tuberculosis and other complaints. It is something of a mockery to commend this treatment to those who have to bring up their children in airless slums, and never more so than in such a summer as that which we have recently enjoyed. We turn, therefore, with interest to what the Medical Officer of Health for Poplar has to say in his report on artificial light treatment, in recommending his Council to purchase a mercury-vapour lamp at the modest cost of some £40. He states that he visited the Infants' Hospital, Vincent Square, where the mercury-vapour lamp has proved invaluable in the treatment of cases of rickets, anæmia, and other wasting conditions which are benefited by natural sunshine. Patients are exposed for intervals gradually increasing from five to thirty minutes, back and front, daily, at a distance of a few feet from the lamp. The eyes are protected during exposure. These lamps are used at many hospitals. At the London, patients sit nude, in a circle, and at a distance of about a yard round the arc light; a screen surrounds them outside, and they wear shades over their eyes. A general light bath can be given at first for fifteen minutes, and finally for two hours a day. We are reminded that the influence of light can be neutralised by a defective diet. Subject to this important consideration, it is pleasant to think that some part of our deficiencies in the matter of sunshine and open air can be made good in this way among the toddlers of our murky urban areas.

### MALARIA IN CORSICA.

NOTWITHSTANDING all that has been learned about malaria, and the wonderful progress that has been made in preventing it, there are still civilised places in which it continues to rage. In Corsica, for instance, so far from diminishing, it is increasing. The French Government allocate considerable sums to dealing with it, but since the money is spent by the Department of Roads and Bridges it is not surprising that the results are meagre. The French member of the Committee for Hygiene of the League of Nations, Professor Léon Bernard, declares that the Government medical service in Corsica is entirely unorganised and, being attached to no particular Ministry, is unable to get reforms carried through. He points to the example of Italy as one which might well be followed. In the course of a generation the number of cases noted annually in Italy has fallen from 20,000 to 2,000. This has been accomplished mainly by careful drainage of the affected areas, and it is clear that what Italy can do France can do. There is no longer any excuse for sitting down under the scourge of malaria. We know how to deal with it, but the first essential is organisation.

### AN AMERICAN VIEW OF SANITARY WORK.

"TIN cans do not breed disease. A dead cat in the street will not cause diphtheria. Even a burning dump will not affect health. An untrapped sink in the open will do no harm. Garbage in the ash-heap never results in typhoid fever. To whitewash the cellar will not prolong life. An uncovered cesspool is a cause of death only by drowning. Bad odours are practically harmless." Thus Dr. Charles V. Chapin, the Superintendent of Health at Providence, Rhode Island, in an address to the American Public Health Association, quoted by Dr. Davies, Bristol's Medical Officer, in his report recently issued. There may be some case for Dr. Chapin's suggestion that offensive trades are best dealt with by the engineering department, and general inspection of rubbish collections and obvious offences requiring no expert knowledge by the police. He recognises that protection of the water supply, extension of sewers, abolition of privies, and in certain cases the control of rats and mosquitoes, constitute nuisance work which is worth while. It is to be assumed that Dr. Chapin uses stimulating language in order to press home his point that too great importance is ascribed to the mere dealing with nuisances to the comparative neglect of that class of work which carries advice in disease to the home, makes research into the real causes and methods of spread of communicable disease, and provides for their necessary isolation and control.

